# Precise diagnosis of intracranial hemorrhage and subtypes using a three-dimensional joint convolutional and recurrent neural network

**DOI:** 10.1007/s00330-019-06163-2

**Published:** 2019-04-30

**Authors:** Hai Ye, Feng Gao, Youbing Yin, Danfeng Guo, Pengfei Zhao, Yi Lu, Xin Wang, Junjie Bai, Kunlin Cao, Qi Song, Heye Zhang, Wei Chen, Xuejun Guo, Jun Xia

**Affiliations:** 1 Department of Radiology, Shenzhen Second People’s Hospital, Shenzhen Second Hospital Clinical Medicine College of Anhui Medical University, Shenzhen, China; 2 Department of Engineering, CuraCloud Corporation, Seattle, WA USA; 3 School of Biomedical Engineering, Sun Yat-Sen University, Guangzhou, Guangdong China; 4 Department of Radiology, Tongji Hospital, Tongji University School of Medicine, Shanghai, China; 5 Department of Radiology, Pingshan District People’s Hospital, Shenzhen, Guangdong China; 6 Department of Radiology, Peking University Shenzhen Hospital, Shenzhen, Guangdong China; 7 Department of Radiology, The First Affiliated Hospital of Shenzhen University, Health Science Center, Shenzhen Second People’s Hospital, Shenzhen, China

**Keywords:** Brain, Intracranial hemorrhage (ICH), Multislice computed tomography, 3D imaging, Algorithms

## Abstract

**Objectives:**

To evaluate the performance of a novel three-dimensional (3D) joint convolutional and recurrent neural network (CNN-RNN) for the detection of intracranial hemorrhage (ICH) and its five subtypes (cerebral parenchymal, intraventricular, subdural, epidural, and subarachnoid) in non-contrast head CT.

**Methods:**

A total of 2836 subjects (ICH/normal, 1836/1000) from three institutions were included in this ethically approved retrospective study, with a total of 76,621 slices from non-contrast head CT scans. ICH and its five subtypes were annotated by three independent experienced radiologists, with majority voting as reference standard for both the subject level and the slice level. Ninety percent of data was used for training and validation, and the rest 10% for final evaluation. A joint CNN-RNN classification framework was proposed, with the flexibility to train when subject-level or slice-level labels are available. The predictions were compared with the interpretations from three junior radiology trainees and an additional senior radiologist.

**Results:**

It took our algorithm less than 30 s on average to process a 3D CT scan. For the two-type classification task (predicting bleeding or not), our algorithm achieved excellent values (≥ 0.98) across all reporting metrics on the subject level. For the five-type classification task (predicting five subtypes), our algorithm achieved > 0.8 AUC across all subtypes. The performance of our algorithm was generally superior to the average performance of the junior radiology trainees for both two-type and five-type classification tasks.

**Conclusions:**

The proposed method was able to accurately detect ICH and its subtypes with fast speed, suggesting its potential for assisting radiologists and physicians in their clinical diagnosis workflow.

**Key Points:**

*• A 3D joint CNN-RNN deep learning framework was developed for ICH detection and subtype classification, which has the flexibility to train with either subject-level labels or slice-level labels.*

*• This deep learning framework is fast and accurate at detecting ICH and its subtypes.*

*• The performance of the automated algorithm was superior to the average performance of three junior radiology trainees in this work, suggesting its potential to reduce initial misinterpretations.*

**Electronic supplementary material:**

The online version of this article (10.1007/s00330-019-06163-2) contains supplementary material, which is available to authorized users.

## Introduction

Intracranial hemorrhage (ICH) is a critical disease that may lead to severe disability or death. It could be caused by various reasons ranging from trauma, vascular disease to congenital development [1]. According to the bleeding location, ICH can be further classified as epidural hemorrhage (EDH), subdural hemorrhage (SDH), subarachnoid hemorrhage (SAH), cerebral parenchymal hemorrhage (CPH), and intraventricular hemorrhage (IVH) [2, 3]. The degrees of severity and interventions vary with bleeding types [4].

Computed tomography (CT) is a well-known non-invasive and effective imaging approach to detect ICH [1]. Hemorrhage can be recognized on non-contrast CT since blood has slightly higher density (Hounsfield unit, HU) than other brain tissues but lower than that of bones [5]. The accurate diagnosis of bleeding becomes critical for clinicians to take clinical interventions [6]. In addition, evaluation of head CT is often needed for patients at emergency departments after working hours. In most clinical centers, initial interpretations of head CT is usually provided by junior radiologists, radiology trainees, or emergency physicians in order to give necessary care to clinically significant patients. The initial interpretations will be reviewed later by senior or more-experienced radiologists. Several studies have confirmed that discrepancies exist between the initial and final interpretations and some misinterpretations might even cause clinical consequences [7–10]. Among these studies, Strub et al focused mainly on the misinterpretation of ICH between overnight residents and staff neuroradiologists [10]. It was reported that ICH accounted for 13.6% (141/1037) of the discrepancies and the most common subtypes of misidentified ICH were SDH and SAH, occurring in 39% and 33% of the cases, respectively [10]. Therefore, an automated triage system for accurate ICH detection is desirable to reduce the rate of misdiagnosis.

Recently, artificial intelligence (AI) has shown great promise in the medical imaging domain [11–16]. Among these, some studies have made attempts to detect abnormalities in head CT including ICH using deep learning/machine learning methods [17–22]. Prevedello et al demonstrated the application of a simple deep learning algorithm to detect critical test findings for head CT using a small dataset with 76 acute ICH cases [23]. Li et al reported high diagnostic value (100% sensitivity and 92% specificity) for SAH detection by applying a supervised machine learning algorithm to 129 subjects with suspected SAH [18]. A more recent study by Chang et al applied a hybrid convolutional neural network (CNN) using slice slabs on a dataset containing 10,159 training CT scans and 862 testing CT scans from a single institution for ICH detection and quantification [22]. However, this large dataset contains a low amount of ICH-positive cases (901 and 82 for training and testing, respectively) and not all ICH subtypes were analyzed in this study. Another recent study by Chilamkurthy et al used deep learning for automatic detection of critical findings in head CT scans, including ICH with 4304 scans [20]. A two-stage approach was employed, in which a 2D CNN was used to obtain slice-level confidence and random forest was then adopted to predict subject-level probability. It should be noted that the methods above were based on 2D or slice slabs, and the subject-level prediction was then obtained by iterating through all slices and combining slice-level results with post-processing. Slice-level labels were required for training. Attempts have been made by Arbabshirani et al to apply a 3D CNN-based approach to detect ICH [24], in which a simple CNN network with five convolutional layers and two fully connected layers was adopted and only subject-level labels were used as ground truths for training. The performance of this plain 3D CNN seemed improvable (AUC = 0.846, sensitivity = 0.73, and specificity = 0.80 at the chosen operating point [24]). It remains unknown whether such straightforward approaches (2D, hybrid, or simple 3D) are able to generate reliable predictions.

This study aimed at developing a novel framework for automated and accurate ICH detection. The framework was built based upon a relatively large size of datasets collected from multiple centers with varieties of CT scanners. It seamlessly integrated CNN and recurrent neural network (RNN) in which CNN was used to extract useful features from image slices while RNN was employed to consider inter-slice dependency context. Our framework is an end-to-end trainable network with the flexibility for training under two different levels of annotation details: (1) only ground truths of subjects (i.e., labels for the whole scans) are available and (2) ground truths for each of the slices in the scans are available. The first scenario requires fewer annotation efforts, which may be preferred if the time for annotation is limited or slice-level annotation is thought to be less reliable. The second scenario demands more annotation efforts, yet provides detailed hemorrhage localization information that may benefit algorithm training. We evaluated and compared the performance of our proposed algorithm under both settings. A visualization mechanism was also proposed to provide visual evidence of detection, which does not require any manual delineation of bleeding areas for training. We further demonstrated the potential usefulness of our framework by comparing the performance of our algorithm with that of two groups of head CT interpreters with different levels of experience.

## Materials and methods

### Study cohort

**Fig. 1:**
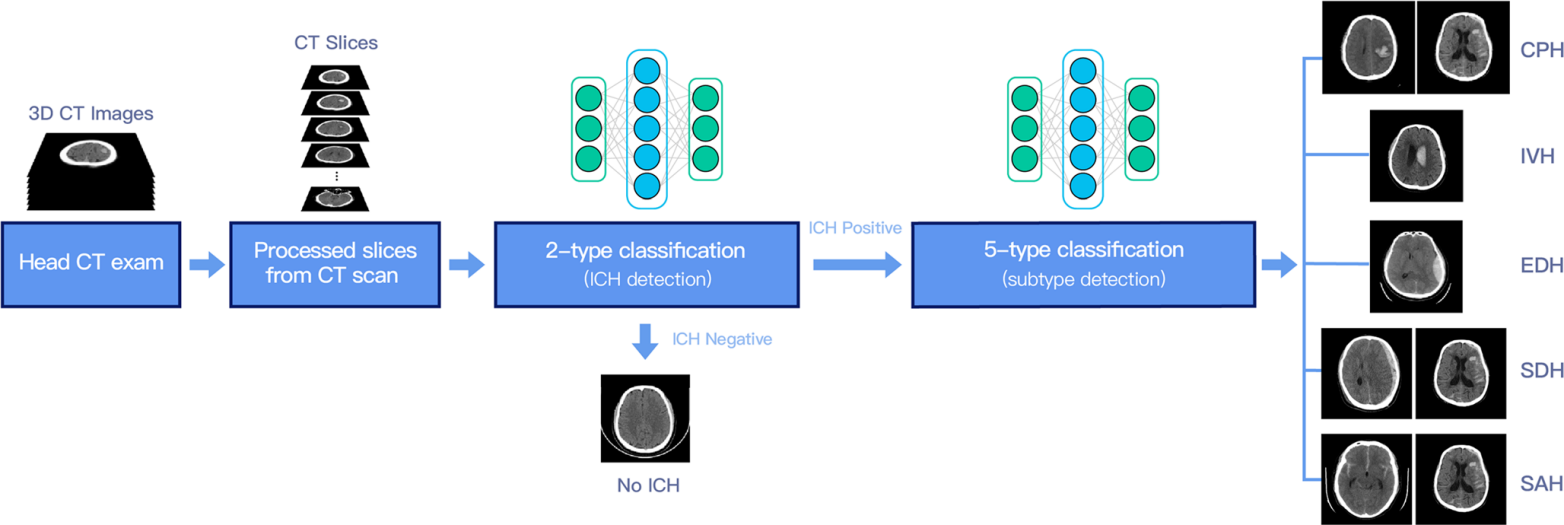
Demonstration of ICH and its subtype prediction workflow. Given processed CT images, two-type classification was first applied to predict if a subject showed ICH. If a subject was predicted to be ICH-positive by our algorithm, we further applied five-type classification to determine which (one or more) of the five subtypes of ICH this subject had

This retrospective study was approved by the ethics committees of three participating hospitals (hospital A, hospital B, and hospital C). Head CT scans from 3129 subjects were initially collected, with 2102 from hospital A, 511 from hospital B, and 516 from hospital C. All subjects were from the Asian population. The detailed study cohort design is described in Supplementary Material. After careful slice-wise review and annotation by three independent experienced radiologists (with 10, 12, and 16 years’ experience in interpreting head CT scans, respectively), 293 cases were excluded from further analysis due to incomplete information or serious imaging artifacts. The remaining 2836 cases were finally used in our study, including 1836 subjects with ICH and 1000 normal subjects. We intentionally kept such a high ICH prevalence (65%) in this dataset to ensure that there were sufficient positive samples to benefit the learning process of the algorithms as well as to effectively evaluate our algorithms with sufficient positive and negative samples. Table 1 shows the demographic characteristics of these subjects. The differences of patient age and sex distribution between the non-ICH group and ICH group were tested using ANOVA and *χ*^2^ test, respectively, with *p* values reported in Table 1. Statistical significance for both age and sex distributions between these two groups is consistent with previous findings that the incidence ratio of ICH tends to be higher in males and in more aged subjects [25–29]. Subjects in the ICH group were further categorized into five subtypes according to the location of ICH on both the slice-level and the subject-level: CPH, IVH, SDH, EDH, and SAH. It is possible for some subjects with ICH presence to have more than one subtypes (i.e., mixed subtypes). Table 2 shows the inter-rater annotation agreement among the three radiologists. The majority vote of these three senior radiologists’ annotations (slice-level and subject-level bleeding as well as subtypes) was used as the gold standard. Examples of scan slices used in this study are shown in Fig. 1.

**Table 1:** Demographic information of subjects used in this study

	Non-ICH	ICH	*p* value
*n*	1000	1836	–
Age (years)*	41.58 ± 15.26 (2–82)	53.91 ± 16.51 (1–98)	< 0.001
Sex (male:female)	448:552	1195:641	< 0.001

*Age reported as mean ± standard deviation (minimum–maximum)

**Table 2:** Subject-level and slice-level scoring variability assessment of three radiologists on the diagnosis of ICH and five subtypes

		R1 and R2		R2 and R3		R1 and R3		*K*
		*p* (%)	*κ*	*p* (%)	*κ*	*p* (%)	*κ*	
ICH	Subject	100	1.00	99	0.99	99	0.99	0.99
	Slice	93	0.83	96	0.91	92	0.80	0.85
CPH	Subject	91	0.77	95	0.87	91	0.77	0.80
	Slice	95	0.85	97	0.92	95	0.84	0.87
SAH	Subject	86	0.70	87	0.73	85	0.68	0.71
	Slice	89	0.65	91	0.74	89	0.62	0.67
EDH	Subject	98	0.85	98	0.83	97	0.80	0.82
	Slice	99	0.79	99	0.82	99	0.73	0.78
SDH	Subject	94	0.78	94	0.78	93	0.72	0.76
	Slice	97	0.74	97	0.78	95	0.64	0.72
IVH	Subject	87	0.72	94	0.87	88	0.74	0.78
	Slice	93	0.71	97	0.88	94	0.73	0.78

*R*, radiologist; *p*, percentage agreement rate

*κ*, Cohen’s kappa coefficient, a statistic that measures inter-rater agreement and is more robust than percent agreement rate. A number greater than 0.6 indicates substantial agreement, while greater than 0.8 indicates almost perfect agreement

*Κ*, Fleiss’ kappa coefficient, a statistic that measures the reliability of agreement between multiple raters. A number greater than 0.6 indicates substantial agreement, while greater than 0.8 indicates almost perfect agreement

### Non-contrast CT imaging protocol

Head CT images used in this study were acquired by scanners from different manufacturers. The scanning parameters were different among these three institutions, with details listed in Supplementary Table 1.

### Data pre-processing

To feed the data for training, we first performed pre-processing of the original CT images with the following steps. All image slices were resampled to 512 × 512 pixels if necessary and then downsampled to 256 × 256 pixels to reduce GPU memory usage. The original slice number of each scan was kept. To better account for the high dynamic intensity range while preserving the details for different objects of interest, we chose three different intensity windows to normalize images, with details described in Supplementary Material.

### Prediction models and workflow

To reduce redundancy, hereinafter, we refer to the scenario that only subject-level ground truths were used in training as *Sub-Lab*, and the scenario that subject-level labels together with slice-level labels were used in training as *Sli-Lab*. Furthermore, we refer to the task of predicting whether a subject and its slices contain bleeding or not as a two-type classification, while the task of predicting the bleeding subtype(s) of an ICH-positive subject and the associated slices as a five-type classification. Our framework can be used for both two-type and five-type classification under both Sub-Lab and Sli-Lab settings. Specifically, this algorithm is composed of a CNN component followed by a RNN component to mimic how radiologists interpret scans. The CNN component focuses on extracting useful features from image slices. The RNN component makes use of these features and generates the probability of ICH or a subtype. The RNN component is particularly useful for capturing sequential information of features from consecutive slices, adding inter-slice dependency context to boost classification performance (please refer to Supplementary Figure 1 for an illustration of our algorithm; more detailed description can be found in Supplementary Material).

In our prediction workflow, we first carried out two-type classification to determine if ICH was present in a subject. If a subject was predicted to be ICH-positive, five-type classification was performed to decide if this subject belonged to any of the five subtypes. This workflow is demonstrated in Fig. 1.

### Training procedures

**Fig. 2:**
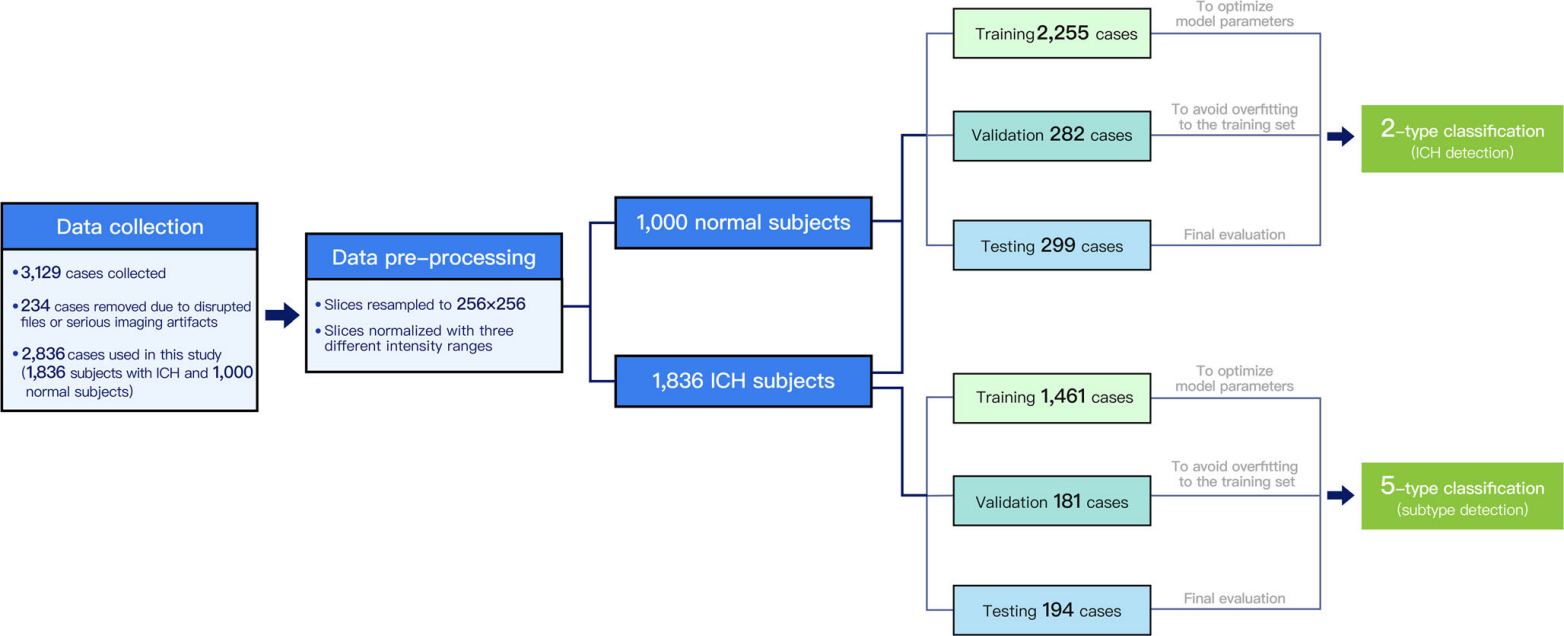
Illustration of training and testing schema of the two-type and five-type classification tasks. Collected data was first pre-processed and then utilized as training, validation, and testing set for two-type and five-type classification tasks

We split the entire subjects randomly into training (80%), validation (10%), and testing set (10%). Data distribution for two-type and five-type classification tasks is shown in Supplementary Table 2. The training set was used to optimize model parameters while the validation set was used to avoid overfitting to the training set. The testing set was reserved for final evaluation of our models. Training and testing schemata are illustrated in Fig. 2. Training for ICH detection (two-type task) and its subtypes (five-type task) was performed under two settings: Sub-Lab and Sli-Lab (more details about the training process are elaborated in Supplemental Material).

### Model visualization

A disadvantage of deep learning models is their lack of transparency and explanability [30, 31]. To improve the explainability of our models, we generated a coarse localization map that highlighted important regions in the image leading to the decision of the algorithm using the Grad-CAM method [31]. The localization map on each slice was generated with our fully trained algorithm, which neither affected the algorithm training process nor required manual annotation of bleeding areas for supervised training. This visualization technique might also be adopted by radiologists as a guidance for interpretation (more details are provided in Supplementary Material).

### Statistical analysis

All statistical analyses were performed using the python package scikit-learn, while statistical plots were generated with matplotlib. We evaluated the performance of algorithms using statistical metrics including accuracy, sensitivity, specificity, F1 score, and area under the curve (AUC). We used 0.5 as the threshold to convert probabilities into binarized class labels, i.e., a probability no smaller than 0.5 was considered ICH-positive and a probability smaller than 0.5 to be ICH-negative.

### Diagnosis from additional radiologists and trainees

We additionally invited three junior radiology trainees and an additional senior radiologist to provide subject-level diagnosis on the 299 CT scans in the testing set for performance comparison with the automated algorithm (more details about these head CT interpreters can be found in Supplementary Material).

## Results

### Two-type classification task

**Fig. 3:**
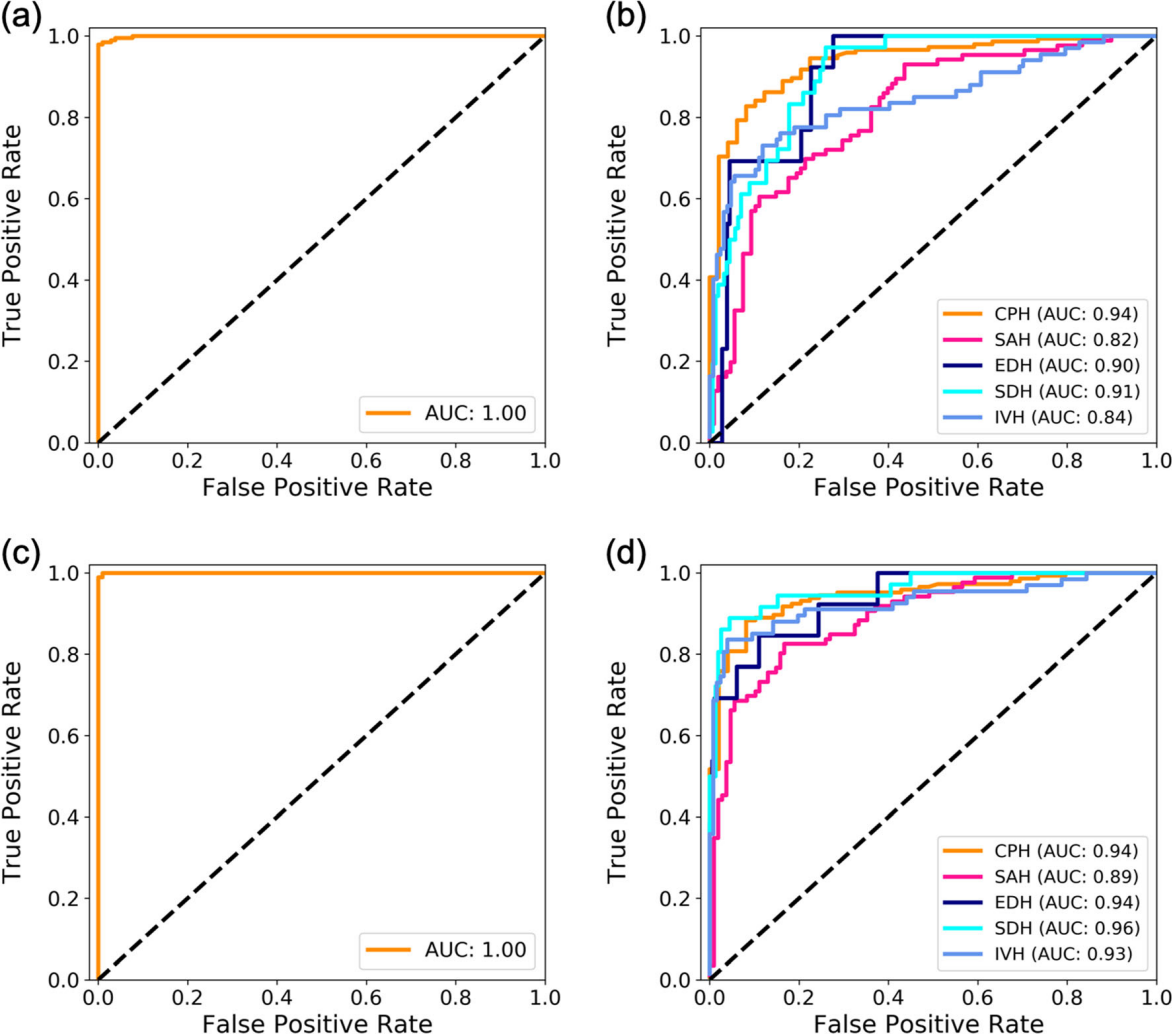
Subject-level ROC curves and AUC results for two-type and five-type classification tasks. **a**, **b** two-type and five-type results for algorithm trained with only subject-level labels. **c**, **d** two-type and five-type results for algorithm trained with both subject-level and slice-level labels. The dashed black line shows the diagonal between coordinates (0, 0) and (1, 1). AUC is shown in the legend of each plot

We evaluated the performance of our two-type classification in the testing set, which contained 299 subjects with 8007 slices in total. Sixty-five percent of the subjects and 23% of the slices were ICH-positive, respectively. The subject-level performance of our algorithm is reported in Table 3 and Fig. 3. Algorithms trained under both Sub-Lab and Sli-Lab settings achieved excellent values (≥ 0.98), with minimal differences across all evaluation metrics between these two settings. The results of additional experiments, including the performance comparison of our models with baseline models, are provided in Supplementary Material.

**Table 3:** Subject-level performance of the automated algorithm, three junior radiology trainees, and a senior radiologist on two-type and five-type classification tasks

		Accuracy	Sensitivity	Specificity	F1 score	AUC
ICH	Model (Sub-Lab)	0.99	0.98	0.99	0.99	1.00
	Model (Sli-Lab)	0.99	0.99	0.99	0.99	1.00
	JRT 1	0.94	0.91	1.00	0.95	0.96
	JRT 2	0.97	0.97	0.97	0.98	0.97
	JRT 3	0.97	0.95	1.00	0.97	0.97
	JRT (*x̅* ± *s*)	0.96 ± 0.02	0.94 ± 0.03	0.99 ± 0.02	0.96 ± 0.02	0.97 ± 0.01
	SR	1.00	1.00	1.00	1.00	1.00
CPH	Model (Sub-Lab)	0.88	0.90	0.82	0.92	0.94
	Model (Sli-Lab)	0.90	0.92	0.83	0.93	0.94
	JRT 1	0.84	0.79	1.00	0.88	0.89
	JRT 2	0.92	0.92	0.90	0.94	0.91
	JRT 3	0.87	0.86	0.90	0.91	0.88
	JRT (*x̅* ± *s*)	0.88 ± 0.04	0.86 ± 0.07	0.93 ± 0.06	0.91 ± 0.03	0.89 ± 0.02
	SR	0.95	0.98	0.86	0.97	0.92
SAH	Model (Sub-Lab)	0.75	0.65	0.82	0.7	0.82
	Model (Sli-Lab)	0.83	0.69	0.94	0.78	0.89
	JRT 1	0.62	0.19	0.96	0.30	0.57
	JRT 2	0.81	0.58	1.00	0.74	0.79
	JRT 3	0.65	0.27	0.95	0.40	0.61
	JRT (*x̅* ± *s*)	0.69 ± 0.10	0.35 ± 0.21	0.97 ± 0.03	0.48 ± 0.23	0.66 ± 0.12
	SR	0.96	0.95	0.96	0.95	0.96
EDH	Model (Sub-Lab)	0.92	0.69	0.94	0.55	0.90
	Model (Sli-Lab)	0.96	0.69	0.98	0.72	0.94
	JRT 1	0.97	0.54	1.00	0.73	0.77
	JRT 2	0.98	0.77	1.00	0.87	0.88
	JRT 3	0.96	0.85	0.97	0.73	0.91
	JRT (*x̅* ± *s*)	0.97 ± 0.01	0.72 ± 0.16	0.99 ± 0.02	0.78 ± 0.08	0.85 ± 0.07
	SR	0.99	0.92	1.00	0.96	0.96
SDH	Model (Sub-Lab)	0.87	0.61	0.93	0.64	0.91
	Model (Sli-Lab)	0.94	0.86	0.96	0.84	0.96
	JRT 1	0.88	0.53	0.96	0.62	0.75
	JRT 2	0.94	0.75	0.99	0.83	0.87
	JRT 3	0.91	0.50	1.00	0.67	0.75
	JRT (*x̅* ± *s*)	0.91 ± 0.03	0.59 ± 0.14	0.98 ± 0.02	0.71 ± 0.11	0.79 ± 0.07
	SR	0.98	0.94	0.99	0.96	0.97
IVH	Model (Sub-Lab)	0.84	0.66	0.94	0.74	0.84
	Model (Sli-Lab)	0.91	0.84	0.95	0.87	0.93
	JRT 1	0.83	0.57	0.97	0.70	0.77
	JRT 2	0.92	0.82	0.98	0.88	0.90
	JRT 3	0.88	0.72	0.97	0.81	0.84
	JRT(*x̅* ± *s*)	0.88 ± 0.05	0.70 ± 0.13	0.97 ± 0.01	0.80 ± 0.09	0.84 ± 0.07
	SR	0.96	1.00	0.94	0.94	0.97

Sub-Lab, only subject-level labels were available and used in the training process. Sli-Lab, slice-level labels were available; thus, both slice-level and subject-level labels were used in the training process

*JRT*, junior radiology trainee; *SR*, senior radiologist

*x̅* ± *s*, mean ± standard deviation

### Five-type classification task

We evaluated the performance of our five-type classification of 194 subjects with ICH. CPH showed the highest proportion of positive cases among the five subtypes, while EDH the lowest (CPH > SAH > IVH > SDH > EDH; see Supplementary Table 2 for detailed numbers). Our algorithm achieved > 0.8 AUC and > 0.8 specificity across all subtypes under both Sub-Lab and Sli-Lab settings. Three important observations can be made based on the sensitivity metric. Firstly, CPH was the best-performed subtype, with sensitivity values higher than 0.9 for both Sub-Lab and Sli-Lab settings. Secondly, the sensitivity of the model trained under Sub-Lab was consistently lower than that trained under Sli-Lab for all five subtypes. This may indicate that slice-level information can be more important for subtype classification than for two-type classification task. Thirdly, even for the model trained with slice-level labels, the sensitivity for SAH and EDH was only 0.69, notably lower than that for the other three subtypes. The low sensitivity score of SAH may be due to the difficulty for detection as it has been considered as the most challenging subtype to diagnose [10], while the low sensitivity score for EDH may be mainly caused by the extremely low amount of positive cases: only 6.4% (94/1461) of the subjects and 1.9% (758/39,278) of the slices are EDH-positive. Additional experiments and results are described in Supplementary Material.

### Visualization of results

**Fig. 4:**
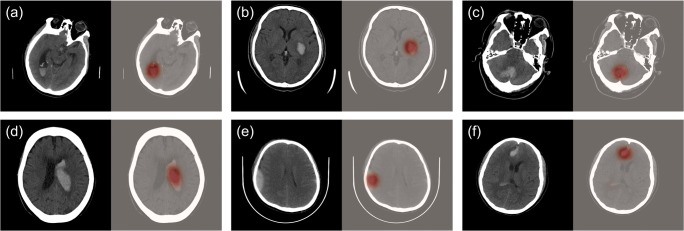
Examples of regions that our algorithm paid most attention to when making decisions using the Grad-CAM approach. **a**–**f** Results for slices with different bleeding locations and different sizes of bleeding areas. Red means high importance while gray means low importance

In addition to statistical evaluations of our models, we used the Grad-CAM method [31] on the model trained under Sli-Lab to generate heatmaps to visually check if our models made decisions based upon reasonable regions. Six examples from the testing set are shown in Fig. 4, where red regions indicated highly important areas for decision making and gray indicated low importance. These heatmaps elucidated that our algorithm paid most attention to the bleeding areas and ignored regions without hemorrhage as expected.

### Performance comparison with radiologists and trainees

**Fig. 5:**
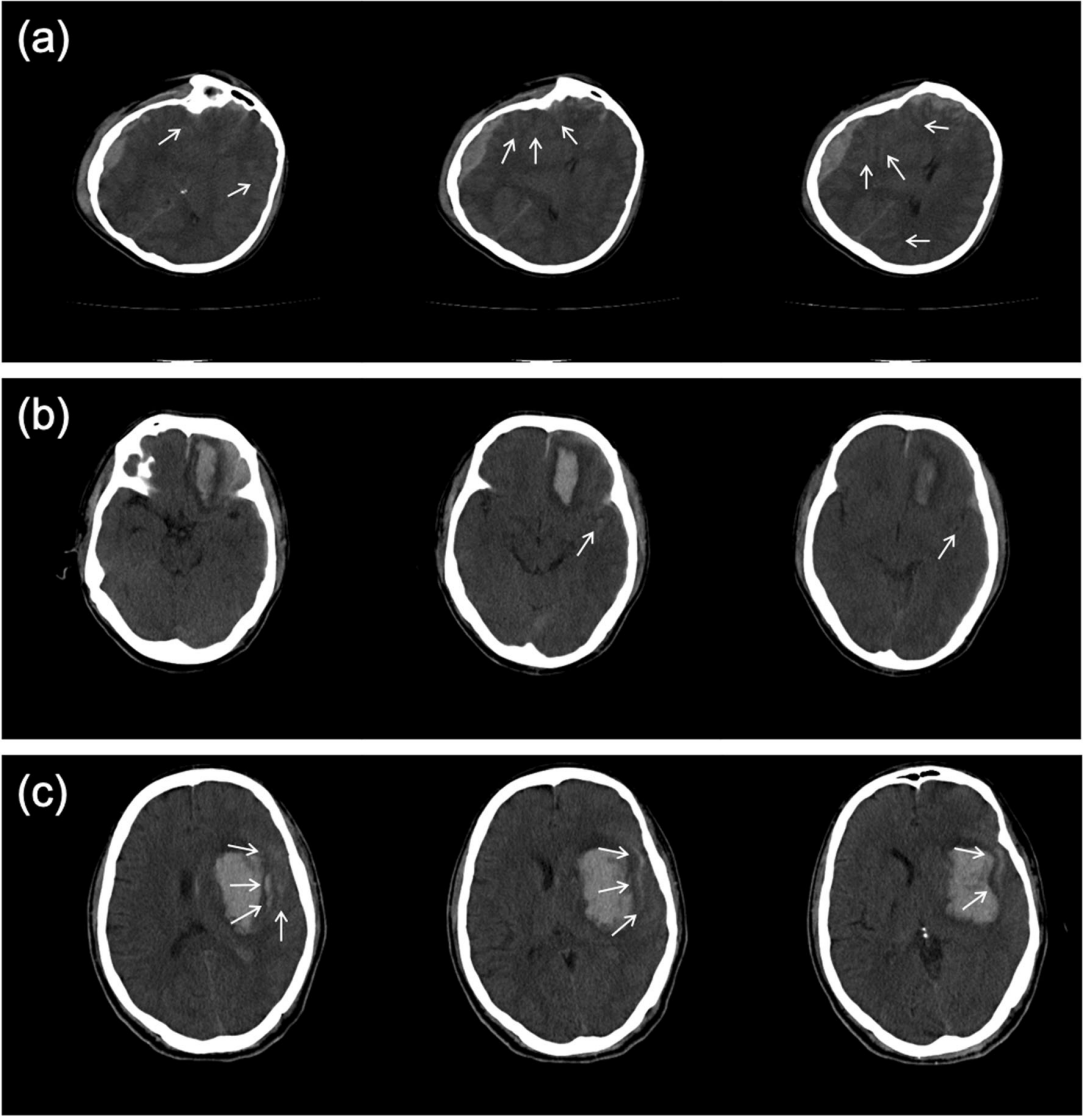
Representative examples of SAH-positive cases that were misdiagnosed by all three junior radiology trainees but correctly predicted by our algorithm. **a**–**c** Three consecutive slices around the SAH hemorrhage loci for each example. The white arrows point to the SAH hemorrhage loci confirmed by the senior radiologist

We additionally compared the results of our models with the interpretations from three junior radiology trainees and an additional senior radiologist using the testing set. Table 3 shows the subject-level performance comparison. For simplicity, in the following, we only focus on the algorithm trained under Sli-Lab due to its better performance. In the two-type classification task, the senior radiologist classified all subjects correctly, while the junior radiology trainees misdiagnosed 12 (4%) cases (11 false negatives and 1 false positive) on average. In comparison, our algorithm under Sli-Lab only incorrectly predicted 2 (< 1%) CT scans (1 false positive and 1 false negative) when training with slice-level labels. More importantly, our algorithm correctly classified on average 10.7 (17, 10, and 5 for the three junior radiology trainees, respectively) ICH-positive cases that the junior radiology trainees misdiagnosed. For the five-type classification task, the senior radiologist performed generally the best across all five subtypes, especially for the sensitivity metric. The sensitivity of our algorithm was higher than the average performance of the junior radiology trainees for CPH, SAH, SDH, and IVH. Even for EDH with an extremely low amount of positive cases (6.4%), the sensitivity of our algorithm was merely 0.03 lower than the average performance of the junior radiology trainees. SAH has been considered as the most difficult subtype to diagnose [10]. Indeed, it showed the most notable discrepancy for the sensitivity metric: 0.95 for the senior radiologist, 0.69 for our algorithm, while only 0.35 for the average performance of the junior radiology trainees. Further, our algorithm correctly predicted 11 (13%) SAH cases that none of the three junior radiology trainees were able to interpret correctly. All of these SAH cases have mixed hemorrhage subtypes, making the SAH subtype liable to being overlooked (please see Fig. 5 for three examples). By contrast, there was only one SAH-positive case that all three junior radiology trainees captured but our algorithm failed. We presented this case in Supplementary Material.

## Discussion

In this study, we proposed a joint CNN-RNN deep learning algorithm and a prediction workflow for ICH and its subtypes. The contribution can be summarized at least in the following three aspects. Firstly, to the best of our knowledge, our proposed algorithm was the first end-to-end trainable 3D ICH detection deep learning network that seamlessly integrates CNN and RNN and meanwhile provides the flexibility of training when only subject-level labels are available or slice-level labels are available. Performance comparison with baseline CNN models (Supplementary Material) confirmed that combining the advantages of CNN and RNN indeed improved ICH detection. Secondly, a comprehensive framework for subject-level bleeding and its subtype prediction was proposed using a relatively large size of datasets from multiple centers. Thirdly, in order to provide visual evidence of the detection in our deep learning model, a visualization mechanism was proposed based on our framework and the Grad-Cam approach [31]. It is capable of generating a coarse hemorrhage region in head CT slices using our classification model without manual delineation (segmentation) of bleeding areas for supervised training. Despite that further quantitative performance evaluation is needed, this feature has the potential to be employed by radiologists as a coarse bleeding localization map. In summary, our proposed algorithm could assist detection of ICH and subtypes with high accuracy and may potentially serve as a useful tool to assist diagnosis of ICH.

To improve the reliability of reference standards, this study applied majority voting on the subject-level and slice-level diagnosis from three senior radiologists with over 10 years’ experience in interpreting head CT scans. Slice-level concordance among the three radiologists was generally lower than that on the subject-level: only one kappa value for the subject-level agreement was below 0.7 (0.68), while four on the slice level (0.65, 0.62, 0.67, and 0.64; please refer to Table 2 for details). This observation reflects the difficulty and variation in interpreting slices in head CT, especially for challenging subtypes such as SAH. Less reliability of slice-level diagnosis may be one reason for the preference of only using subject-level labels in the training process without including slice-level information. However, our results showed that adding (less reliable) slice-level labels in the training process was still able to improve the algorithm’s performance, especially by quite a noticeable margin for five-type classification task, indicating the importance of local information for subtype detection.

To further elucidate the potential usefulness of our algorithm, its performance was compared to that of three junior radiology trainees and a senior radiologist. The results showed that its performance was superior to the average performance of the three junior radiology trainees for both the two-type and five-type tasks. SAH has been reported as the most difficult subtype to interpret [10]. In our study, the junior radiology trainees were only able to identify 35% of the subjects with SAH on average. This low sensitivity may be due to the high proportion of SAH cases with blended ICH subtypes in the testing set: 35% (30 out of 86) of the SAH cases had one other subtype present at the same time, while 52% (45 out of 86) had two or more additional subtypes. Mixed subtypes may raise difficulties for diagnosis and may lead to search satisfaction. By contrast, our algorithm not only detected on average 90% (14 out of 16, 21 out of 23, and 45 out of 50 for the three trainees respectively) of the true positives identified by the junior radiology trainees, but also captured another 11 (13%) SAH-positive cases that none of the junior radiology trainees diagnosed correctly, with minimal loss of specificity (0.94 vs 0.97). It is also worth pointing out that it took our algorithm less than 30 s on average to fully process a 3D head CT scan from end to end (namely from scan loading to prediction generation), which is substantially shorter than the reported head CT interpretation time of radiologists (usually more than 5 min [32]).

Our study has several limitations. Firstly, in order to enhance pattern recognition in algorithm training and to carry out performance evaluation with sufficient positive and negative samples, the prevalence of ICH used in our study (65%) was designed to be much higher than that in a real clinical setting (for example, CPH has been reported to have an incidence rate of 25 per 100,000 persons per year [25]). Despite that four reported performance metrics (sensitivity, specificity, F1 score, and AUC) would not be affected by the prevalence in the testing dataset, the accuracy may change with different prevalence levels, which needs further evaluation in a real clinical population. Secondly, all subjects in our study were from the Asian population, which could limit the generalizability of our algorithms. As a next step, it is desirable to expand the current dataset to include populations other than Asian. Thirdly, although the total number of subjects with ICH presence in our dataset was relatively large, the number of subjects with certain subtypes was quite limited, in particular EDH with only 94 cases in the training set, compared with 1367 controls. Since deep learning requires a sufficient amount of data for pattern recognition, lack of training data might have adversely affected the algorithm performance. Finally, the low SAH identification rate of junior radiology trainees may need further investigation and may limit the generalizability of the performance comparison with the automated algorithm. Performance from junior radiology trainees with different training levels may be needed to increase the reliability of the results.

In conclusion, this is one of the early studies that utilized end-to-end trainable 3D deep learning techniques for ICH and subtype detection with a relatively large study cohort. The proposed algorithm was fast and accurate, indicating its potential for assisting less-experienced head CT interpreters such as junior radiology trainees to reduce initial misinterpretations. It would be worthwhile to implement this automated framework in a triage system in a real clinical setting to evaluate its capability of reducing radiologists’ workload and improving efficiency.

## Electronic supplementary material

ESM 1 (DOCX 619 kb)

## Abbreviations

3D: Three-dimensional
AI: Artificial intelligence
AUC: Area under the curve
CNN: Convolutional neural network
CPH: Cerebral parenchymal hemorrhage
CT: Computed tomography
EDH: Epidural hemorrhage
FC: Fully connected
ICH: Intracranial hemorrhage
IVH: Intraventricular hemorrhage
RNN: Recurrent neural network
ROC: Receiver operating characteristic
SAH: Subarachnoid hemorrhage
SDH: Subdural hemorrhage

## References

[1] Heit JJ, Iv M, Wintermark M (2017). Imaging of intracranial hemorrhage. J Stroke.

[2] Bonatti M, Lombardo F, Zamboni GA, Pernter P, Mucelli RP, Bonatti G (2017). Dual-energy CT of the brain: comparison between DECT angiography-derived virtual unenhanced images and true unenhanced images in the detection of intracranial haemorrhage. Eur Radiol.

[3] Qureshi AI, Tuhrim S, Broderick JP, Batjer HH, Hondo H, Hanley DF (2001). Spontaneous intracerebral hemorrhage. N Engl J Med.

[4] Carney N, Totten AM, O’Reilly C (2017). Guidelines for the management of severe traumatic brain injury, Fourth Edition. Neurosurgery.

[5] Nguyen HS, Li L, Patel M, Mueller W (2016). Density measurements with computed tomography in patients with extra-axial hematoma can quantitatively estimate a degree of brain compression. Neuroradiol J.

[6] Elliott J, Smith M (2010). The acute management of intracerebral hemorrhage: a clinical review. Anesth Analg.

[7] Alfaro D, Levitt MA, English DK, Williams V, Eisenberg R (1995). Accuracy of interpretation of cranial computed tomography scans in an emergency medicine residency program. Ann Emerg Med.

[8] Lal NR, Murray UM, Eldevik OP, Desmond JS (2000). Clinical consequences of misinterpretations of neuroradiologic CT scans by on-call radiology residents. AJNR Am J Neuroradiol.

[9] Erly WK, Berger WG, Elizabeth K, Seeger JF, Guisto JA (2002). Radiology resident evaluation of head CT scan orders in the emergency department. AJNR Am J Neuroradiol.

[10] Strub WM, Leach JL, Tomsick T, Vagal A (2007). Overnight preliminary head CT interpretations provided by residents: locations of misidentified intracranial hemorrhage. AJNR Am J Neuroradiol.

[11] Litjens G, Kooi T, Bejnordi BE (2017). A survey on deep learning in medical image analysis. Med Image Anal.

[12] Chen X, Lu Y, Bai J et al (2018) Train a 3D U-Net to segment cranial vasculature in CTA volume without manual annotation. 2018 IEEE 15th International Symposium on Biomedical Imaging (ISBI 2018):559–563

[13] Havaei M, Davy A, Warde-Farley D (2017). Brain tumor segmentation with deep neural networks. Med Image Anal.

[14] Kamnitsas K, Ledig C, Newcombe VFJ (2016). Efficient multi-scale 3D CNN with fully connected CRF for accurate brain lesion segmentation. Med Image Anal.

[15] Avendi MR, Kheradvar A, Jafarkhani H (2016). A combined deep-learning and deformable-model approach to fully automatic segmentation of the left ventricle in cardiac MRI. Med Image Anal.

[16] Shin H-C, Roth H, Gao M (2016). Deep convolutional neural networks for computer-aided detection: CNN architectures, dataset characteristics and transfer learning. IEEE Trans Med Imaging.

[17] Xiao F, Liao CC, Huang KC, Chiang IJ, Wong JM (2010). Automated assessment of midline shift in head injury patients. Clin Neurol Neurosurg.

[18] Li Y-H, Zhang L, Hu Q-M, Li H, Jia F-C, Wu J-H (2011). Automatic subarachnoid space segmentation and hemorrhage detection in clinical head CT scans. Int J Comput Assist Radiol Surg.

[19] Merkow J, Lufkin RB, Nguyen K, Soatto S, Tu Z, Vedaldi A (2017) DeepRadiologyNet: radiologist level pathology detection in ct head images. arXiv preprint arXiv:1711.09313

[20] Chilamkurthy S, Ghosh R, Tanamala S et al (2018) Development and validation of deep learning algorithms for detection of critical findings in head CT scans. arXiv preprint arXiv:1803.05854 10.1016/S0140-6736(18)31645-3 30318264

[21] Titano JJ, Badgeley M, Schefflein J (2018). Automated deep-neural-network surveillance of cranial images for acute neurologic events. Nat Med.

[22] Chang P, Kuoy E, Grinband J (2018). Hybrid 3D/2D convolutional neural network for hemorrhage evaluation on head CT. AJNR Am J Neuroradiol.

[23] Prevedello LM, Erdal BS, Ryu JL (2017). Automated critical test findings identification and online notification system using artificial intelligence in imaging. Radiology.

[24] Arbabshirani MR, Fornwalt BK, Mongelluzzo GJ (2018). Advanced machine learning in action: identification of intracranial hemorrhage on computed tomography scans of the head with clinical workflow integration. npj Digit Med.

[25] van Asch CJ, Luitse MJ, Rinkel GJ, van der Tweel I, Klijn CJ (2010). Incidence, case fatality, and functional outcome of intracerebral haemorrhage over time, according to age, sex, and ethnic origin: a systematic review and meta-analysis. Lancet Neurol.

[26] Araújo JLV, Aguiar UP, Todeschini AB, Saade N, Veiga JCE (2012). Epidemiological analysis of 210 cases of surgically treated traumatic extradural hematoma. Rev Col Bras Cir.

[27] Abdelmalik PA, Ziai WC (2017). Spontaneous intraventricular hemorrhage: when should intraventricular tPA be considered?. Semin Respir Crit Care Med.

[28] Rooij NKD, Linn FHH, Plas JAVD, Algra A, Rinkel GJE (2007). Incidence of subarachnoid haemorrhage: a systematic review with emphasis on region, age, gender and time trends. J Neurol Neurosurg Psychiatry.

[29] Ivamoto HS, Jr HPL, Atallah AN (2016). Surgical treatments for chronic subdural hematomas: a comprehensive systematic review. World Neurosurg.

[30] Zhou B, Khosla A, Lapedriza A, Oliva A, Torralba A (2016) Learning deep features for discriminative localization. 2016 IEEE Conference on Computer Vision and Pattern Recognition (CVPR):2921–2929

[31] Selvaraju RR, Cogswell M, Das A, Vedantam R, Parikh D, Batra D (2017) Grad-CAM: visual explanations from deep networks via gradient-based localization. In: 2017 IEEE International Conference on Computer Vision, pp 618–626

[32] Kim ES, Yoon DY, Lee H-Y (2014). Comparison of emergency cranial CT interpretation between radiology residents and neuroradiologists: transverse versus three-dimensional images. Diagn Interv Radiol.

