# Supplementary material for: Precise diagnosis of intracranial hemorrhage and subtypes using a three-dimensional joint convolutional and recurrent neural network
Source: Eur Radiol. 2019 Apr 30;29(11):6191–201. doi: 10.1007/s00330-019-06163-2 (PMC6795911; doi:10.1007/s00330-019-06163-2)
Supplement: Supplementary file 1 — (DOCX 619 kb) [file 330_2019_6163_MOESM1_ESM.docx]

**Supplementary Material**

**1. Detailed study cohort design**

Non-contrast head CT scans were collected from the three participating hospitals with the following design: (1) the head CT scans of all ICH patients between the date range of November 2013 and May 2018 were collected; (2) a random sample of 1,000 head CT scans were selected from all normal subjects between the date range of November 2013 and May 2018. Exclusion criteria included: (1) subjects who underwent surgery in the brain or had neoplastic lesions; (2) subjects with brain diseases other than ICH, such as brain tumor.

**2. Details of data pre-processing**

To better account for the high dynamic intensity range while preserving the details for different objects of interest, we chose three different intensity (Hounsfield Unit, HU) ranges to normalize images: -50–150, 100–300 and 250–450. The first HU range was chosen to boost the difference between hemorrhage regions and normal tissues. The second HU range was used to emphasize the gradually changed intensity between the inner skull boundary and the skull. The last HU range was selected to reduce the influence of the skull bone on the hemorrhage predictions. As a result, the original one-channel CT image was expanded to three-channels and the values of each channel were then linearly normalized to 0–1.

**3. Details about additional radiologists and trainees**

We additionally invited three junior radiology trainees and an additional senior radiologist to provide subject-level diagnosis for the testing set. At the time of interpretation of the scans, the three junior radiology trainees had completed MD training and were licensed to practice medicine, and they were in the second year of neuroradiology training (out of three years). In the real clinical setting, junior radiology trainees generally serve as initial head CT interpreters in the three participating hospitals. The senior radiologist had ten years’ experience in interpreting head CT scans and was not among the three senior radiologists who participated in the ground truth generation of the dataset.

**4. Details of proposed deep learning architecture**

Our deep learning architecture for ICH and subtype detection consists of a CNN component followed by a RNN component, as shown in Supplementary Figure 1. The CNN component is a sequential model of hierarchical convolutional layers with max-pooling layers and a fully-connected (FC) layer, aiming at extracting useful features from image slices. In our implementation, the convolutional layers are the same as those from VGG16, while the FC layer has a dimension of 256. The RNN component consists of RNN layers and FC layers. This component takes in feature vectors from the FC layer in the CNN component and generates the probability of ICH or subtypes. Owing to the flexibility of our algorithm, training for ICH detection can be easily performed under two scenarios: (1) only subject-level labels are available, referred to as Sub-Lab; (2) slice-level labels are available (then subject-level labels can be generated), referred to as Sli-Lab. Under both scenarios, the raw outputs from the final RNN layer for all slices of a subject (not slice-level probabilities) are averaged as the input for an FC layer (the right grey vertical bar in Supplementary Figure 1(a) and the top grey horizontal bar in Supplementary Figure 1(b)) in the RNN component to generate the subject-level prediction. Under Sli-Lab, in addition to generating the subject-level prediction in the aforementioned way, the raw outputs from the final RNN layer for each slice go through a slice-level FC layer (the right grey vertical bars in Supplementary Figure 1(b)) to generate slice-level predictions, such that both slice-level and subject-level supervision are used in the computation of loss. It is worth pointing out that the parameters in the CNN component, in the RNN layers of the RNN component and in the slice-level FC layer of the RNN component are shared across all slices. In practice, we used 2 layers of bidirectional Gated Recurrent Unit (GRU) [1], a special type of RNN, with a hidden state size of 32 and a dropout rate of 0.2 for each GRU layer. The difference between the 2-type and 5-type classification algorithms is simply the output size of the FC layer for probability generation: 2 for 2-type classification and 5 for 5-type classification. Sigmoid activation function is used to transform the raw outputs from the FC layer to probabilities for both the 2-type and 5-type classification tasks. The algorithms were implemented using the deep learning library PyTorch [2].

**5. Details of training procedures**

In the training process, we applied online augmentation to the training images to reduce overfitting, including rotation (0-180 degrees), scaling (90%-110%), shifting (10%), sheering (10%), as well as horizontal and vertical ﬂipping. Under Sub-Lab, the final loss for optimization is simply the (subject-level) cross-entropy loss *ℓ*_subject_. Under Sli-Lab, the algorithm provides slice-level and subject-level predictions at the same time. In this scenario, the final loss for optimization is defined as a weighted average of slice-level and subject-level cross entropy losses. Given the (averaged) slice-level cross entropy loss as *ℓ*_slice_ and the subject-level cross entropy loss as *ℓ*_subject_, the final loss for parameter optimization is defined as

*ℓ =* ω_slice_ *ℓ*_slice_ + ω_subject_ *ℓ*_subject_,

where ω _slice_ and ω _subject_ are the weights for the subject-level and slice-level losses, respectively. In our experiments, we found that the best performance of our algorithm was achieved when ω_slice_ = 1 and ω_subject_ = 1. We used Adam algorithm [3] to optimize model parameters with a learning rate of 10^-5^. Optimization of model parameters was considered to be converged once the model’s performance on the validation set stopped improving for 50 epochs. Training of all models was performed on an NVIDIA Tesla V100 GPU.

**6. Details of Grad-Cam approach**

To generate the localization map in each slice, given a 2-type classification CNN-RNN model trained under Sli-Lab, we first computed the gradient of the slice-wise score (before sigmoid transformation) of the ICH-positive class $s_{y=1}$ with regards to the feature maps in the final convolution layer (the 5^th^ convolution layer) $A_{ij}^{k}\in\mathbb{R}^{16\times16}$ ($i=1, 2, \ldots, 16;j=1, 2, \ldots, 16;k =1, 2,\ldots, 512$), $\frac{\partial s_{y=1}}{\partial A_{ij}^{k}}$. We then computed the importance weight of any feature map *k* as $\omega_{k}=\frac{1}{Z}\sum_{i} \sum_{j} \frac{\partial s_{y=1}}{\partial A_{ij}^{k}}$, where $Z=\sum_{k} \sum_{i} \sum_{j} \frac{\partial s_{y=1}}{\partial A_{ij}^{k}}$. Next, we computed the normalized heatmap as $\mathcal{H}_{ij}=\frac{1}{Z}ReLU\left( \sum_{k} \omega_{k}A_{ij}^{k} \right)$, where $Z=\sum_{i} \sum_{j} ReLU\left( \sum_{k} \omega_{k}A_{ij}^{k} \right)$ with $ReLU(\cdot)$ being *ReLU* activation function. Finally, we upsampled the obtained 16×16 heatmap to 512×512 to match the original size of the slice.

**7. Additional experiments and results**

We trained several benchmark models to demonstrate the performance of our algorithm using the same dataset, including two 2D models: a 2D VGG16 model [4] and a (2D) GoogleNet [5] (as used in [6]); and one 3D model: a 3D VGG16 model [4]. We also used Adam algorithm [3] to optimize model parameters with a learning rate of 10^-5^ and stopped training once there was no performance improvement for 50 consecutive epochs. For 2D VGG16 and GoogleNet, to convert slice-level probabilities into subject-level prediction, we simply set the subject-level prediction to be positive as long as at least one slice was predicted to be positive. Subject-level and slice-level performance comparisons between our joint CNN-RNN algorithm and the additional benchmark methods were shown in Supplementary Tables 3 and 4, respectively. Our joint CNN-RNN algorithm outperformed or performed as well as the benchmark models in almost all metrics for both the 2-type and 5-type classification tasks at both the slice and the subject levels. Importantly, for detection of challenging subtypes such as SAH, our joint algorithm exhibited a remarkable boost in both the sensitivity and specificity metrics. Additionally, in order to further explore the usefulness of slice-level labels, we trained a 3D CNN-RNN model under Sub-Lab but only replaced the initialization weights from ImageNet to the ones obtained from 2D VGG16 model above. The results showed that its performance for 5-type classification task was notably better than that of Sub-Lab CNN-RNN model (initialized with ImageNet weights) and was actually almost as good as the Sli-Lab CNN-RNN model (please refer to Supplementary Table 3 for detailed numbers). This indicates that detailed slice-level localization information could be very important for the convolution layers of the network.

**8. A representative SAH case predicted incorrectly by our algorithm**

There was only one SAH-positive case that was diagnosed correctly by all three junior radiology trainees but not by our algorithm trained with slice-level labels. The four consecutive slices with SAH presence for this subject are shown in Supplementary Figure 2. Probability predictions of SAH for these 4 slices are 42.6%, 36.1%, 42.8% and 28.8% respectively, and 25.5% for the subject. It can be seen that the hemorrhage locus is adjacent to the cranium, which may interfere signals from the hemorrhage locus and cause confusion to the algorithm.


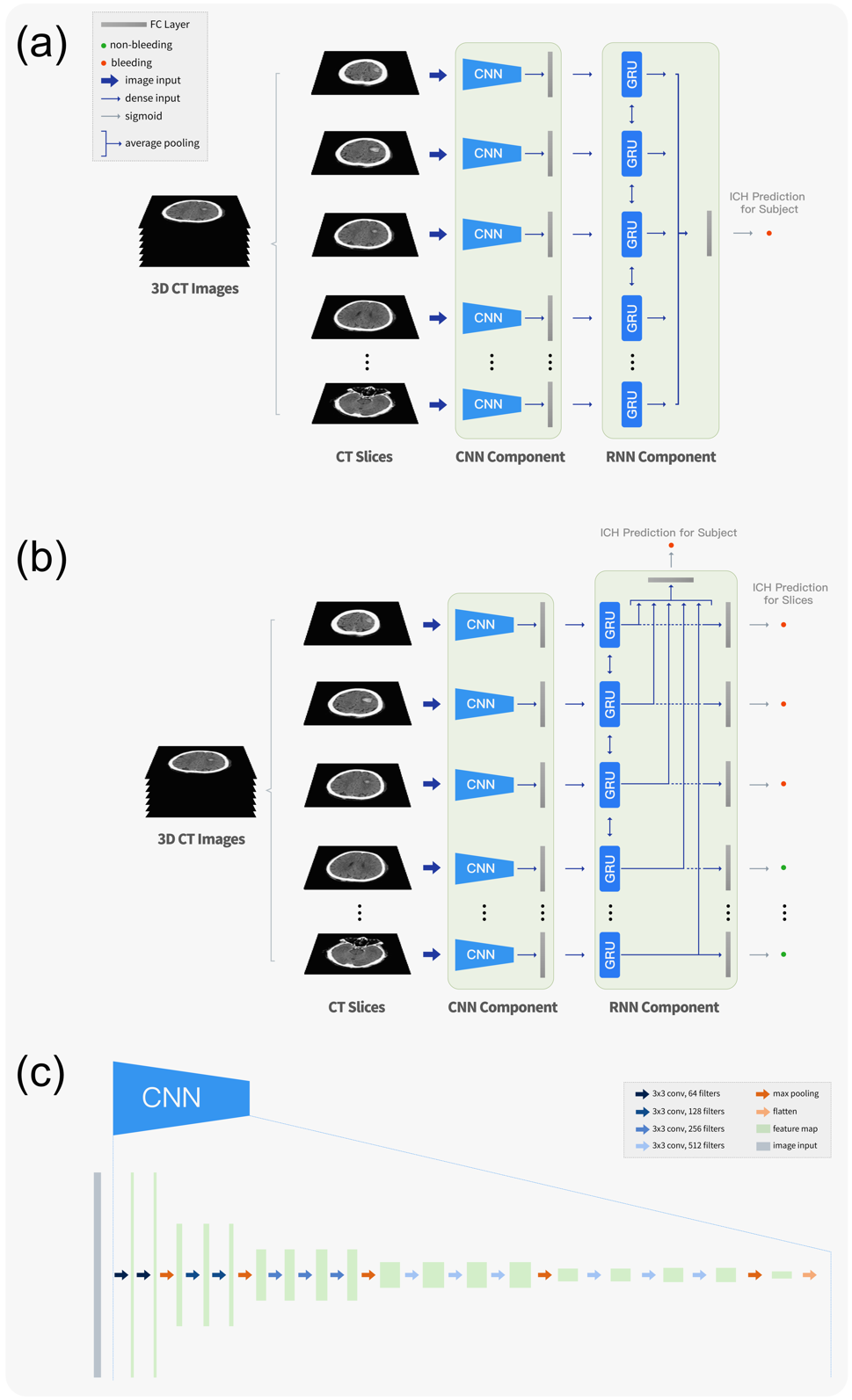


Supplementary Figure 1. Illustration of the deep learning algorithm used in this study. The model contains two major components: the CNN component and the RNN component. The CNN component consists of convolution layers, max pooling layers and a FC layer. The CNN component is designed to generate features from the CT images. The RNN component consists of RNN layers and FC layers. It is used to take in features from the CNN component and generate ICH/subtype probabilities for each slice and/or the subject. (a) Illustration of the algorithm when only subject-level ground truths are available. In this scenario, the RNN component only contains one FC layer that converts the max/average pooling of the output vectors from the final RNN layer into subject-level probabilities. (b) Illustration of the algorithm when slice-level ground truths are available. In this scenario, there are two types of FC layers in RNN component: one FC layer that generates subject-level probabilities, same as in (a); another FC layer that converts the output vectors from the final RNN layer into slice-level probabilities. (c) Detailed CNN structure (VGG16) used in our study.


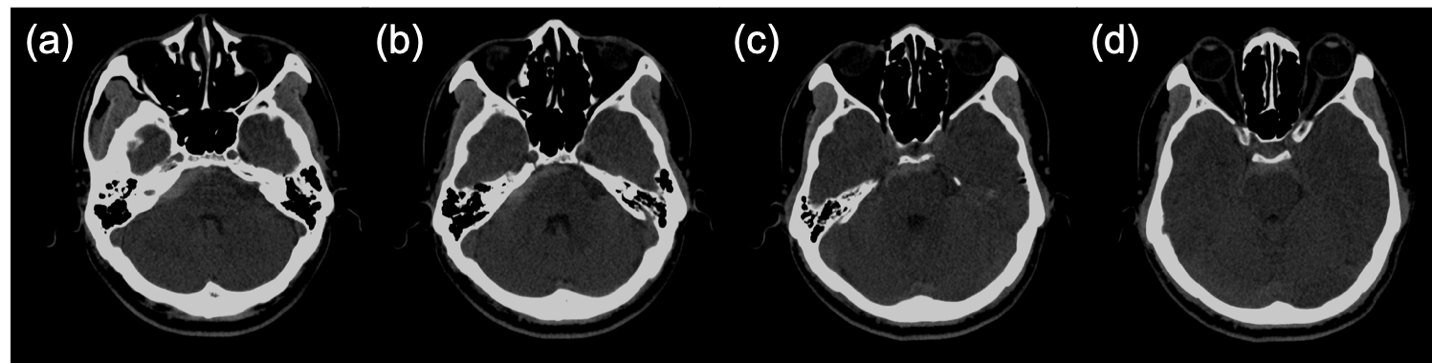


Supplementary Figure 2. Illustration of an SAH-positive case that was diagnosed correctly by all three junior radiology trainees but not by our algorithm CNN-RNN (Sli-Lab). (a)-(d) Four consecutive slices around the hemorrhage locus.

Supplementary Table 1. Detailed non-contrast CT imaging protocol information for three hospitals in our study.

|  | Hospital A | Hospital B | Hospital C |
| --- | --- | --- | --- |
| Manufacturer | SIEMENS | TOSHIBA,  GE MEDICAL SYSTEMS,  SIMENS | GE MEDICAL SYSTEMS |
| Scanner Model | SOMATOM Definition AS,  Emotion 16,  SOMATOM Definition Flash | Aquilion,  Discovery CT750 HD,  SOMATOM Definition Flash,  Revolution CT,  SOMATOM PLUS 4 | Optima CT680 Series,  BrightSpeed,  Revolution CT,  LightSpeed VCT |
| Slice Thickness (mm) | 1--8 | 0.625--10 | 5 |
| Spacing Between Slices (mm) | 1--9.4 | 0.1--10 | 5 |
| Number of slices | 15--80 | 18--60 | 20--50 |
| Exposure time (ms) | 500--2000 | 500--1000 | 500--2500 |

Supplementary Table 2. Data distribution for 2-type and 5-type classification tasks.

|  | Training | | Validation | | Testing | |
| --- | --- | --- | --- | --- | --- | --- |
|  | Subject | Slice | Subject | Slice | Subject | Slice |
| ICH | 2255  (1461:794) | 60995  (39278:21717) | 282  (181:101) | 7619  (4838:2781) | 299  (194:105) | 8007  (5208:2799) |
| CPH | 1461  (977:484) | 39278  (6766:32512) | 181  (127:54) | 4838  (927:3911) | 194  (145:49) | 5208  (982:4426) |
| IVH | 1461  (466:995) | 39278  (3964:35314) | 181  (53:128) | 4838  (498:4340) | 194  (67:127) | 5208  (583:4625) |
| EDH | 1461  (94:1367) | 39278  (758:38520) | 181  (13:168) | 4838  (89:4749) | 194  (13:181) | 5208  (108:5100) |
| SDH | 1461  (219:1242) | 39278  (2086:37192) | 181  (22:159) | 4838  (184:4654) | 194  (36:158) | 5208  (422:4786) |
| SAH | 1461  (558:903) | 39189  (5629:33649) | 181  (71:110) | 4838  (663:4175) | 194  (86:108) | 5208  (811:4397) |

Values shown in the format: total(positive:negative)

Supplementary Table 3. Comparison of subject-level performance among our joint CNN-RNN models (under three training settings), 2D VGG16, 3-D VGG16 and GoogleNet (2D) on 2-type and 5-type classification tasks.

|  | | Accuracy | Sensitivity | Specificity | F1 score | AUC |
| --- | --- | --- | --- | --- | --- | --- |
| ICH | CNN-RNN (Sub-Lab) | 0.99 | 0.98 | 0.99 | 0.99 | 1.00 |
|  | CNN-RNN (Sub-Lab) + pw^*^ | 0.99 | 0.98 | 0.99 | 0.99 | 1.00 |
|  | CNN-RNN (Sli-Lab) | 0.99 | 0.99 | 0.99 | 0.99 | 1.00 |
|  | 2D VGG16 | 0.99 | 0.98 | 0.99 | 0.99 | 0.99 |
|  | 3D VGG16 | 0.94 | 0.95 | 0.93 | 0.96 | 0.98 |
|  | GoogleNet | 0.99 | 0.98 | 1.00 | 0.99 | 0.99 |
| CPH | CNN-RNN (Sub-Lab) | 0.88 | 0.90 | 0.82 | 0.92 | 0.94 |
|  | CNN-RNN (Sub-Lab) + pw | 0.89 | 0.90 | 0.84 | 0.92 | 0.93 |
|  | CNN-RNN (Sli-Lab) | 0.90 | 0.92 | 0.83 | 0.93 | 0.94 |
|  | 2D VGG16 | 0.91 | 0.92 | 0.86 | 0.94 | 0.89 |
|  | 3D VGG16 | 0.81 | 0.87 | 0.63 | 0.87 | 0.81 |
|  | GoogleNet | 0.87 | 0.91 | 0.76 | 0.91 | 0.83 |
| SAH | CNN-RNN (Sub-Lab) | 0.75 | 0.65 | 0.82 | 0.7 | 0.82 |
|  | CNN-RNN (Sub-Lab) + pw | 0.81 | 0.70 | 0.90 | 0.76 | 0.88 |
|  | CNN-RNN (Sli-Lab) | 0.83 | 0.69 | 0.94 | 0.78 | 0.89 |
|  | 2D VGG16 | 0.60 | 0.42 | 0.74 | 0.48 | 0.58 |
|  | 3D VGG16 | 0.75 | 0.63 | 0.84 | 0.69 | 0.84 |
|  | GoogleNet | 0.61 | 0.45 | 0.73 | 0.51 | 0.59 |
| EDH | CNN-RNN (Sub-Lab) | 0.92 | 0.69 | 0.94 | 0.55 | 0.90 |
|  | CNN-RNN (Sub-Lab) + pw | 0.96 | 0.69 | 0.98 | 0.72 | 0.93 |
|  | CNN-RNN (Sli-Lab) | 0.96 | 0.69 | 0.98 | 0.72 | 0.94 |
|  | 2D VGG16 | 0.95 | 0.69 | 0.97 | 0.64 | 0.83 |
|  | 3D VGG16 | 0.93 | 0.38 | 0.97 | 0.43 | 0.78 |
|  | GoogleNet | 0.93 | 0.69 | 0.95 | 0.58 | 0.82 |
| SDH | CNN-RNN (Sub-Lab) | 0.87 | 0.61 | 0.93 | 0.64 | 0.91 |
|  | CNN-RNN (Sub-Lab) + pw | 0.95 | 0.86 | 0.97 | 0.86 | 0.95 |
|  | CNN-RNN (Sli-Lab) | 0.94 | 0.86 | 0.96 | 0.84 | 0.96 |
|  | 2D VGG16 | 0.94 | 0.86 | 0.96 | 0.84 | 0.91 |
|  | 3D VGG16 | 0.83 | 0.42 | 0.92 | 0.48 | 0.83 |
|  | GoogleNet | 0.95 | 0.81 | 0.99 | 0.87 | 0.90 |
| IVH | CNN-RNN (Sub-Lab) | 0.84 | 0.66 | 0.94 | 0.74 | 0.84 |
|  | CNN-RNN (Sub-Lab) + pw | 0.89 | 0.78 | 0.95 | 0.83 | 0.93 |
|  | CNN-RNN (Sli-Lab) | 0.91 | 0.84 | 0.95 | 0.87 | 0.93 |
|  | 2D VGG16 | 0.60 | 0.52 | 0.64 | 0.47 | 0.58 |
|  | 3D VGG16 | 0.79 | 0.55 | 0.91 | 0.64 | 0.82 |
|  | GoogleNet | 0.57 | 0.52 | 0.59 | 0.45 | 0.56 |

Sub-Lab: only subject-level labels are available and used in the training process

Sli-Lab: slice-level labels are available thus both slice-level and subject-level labels are used in the training process

^*^CNN-RNN (Sub-Lab) + pw: The pretrained-weights (pw) from the 2D VGG16 model were used as initialization for CNN-RNN network under Sub-Lab. The only difference between the two models is the weight initialization trick: ImageNet weights were used for initialization in CNN-RNN (Sub-Lab) but the weights from the 2D VGG16 model were used for initialization in CNN-RNN (Sub-Lab) + pw.

Supplementary Table 4. Comparison of slice-level performance among our joint CNN-RNN model (Sli-Lab, trained with slice-level labels), 2D VGG16 and GoogleNet (2D) on 2-type and 5-type classification tasks.

|  | | Accuracy | Sensitivity | Specificity | F1 score | AUC |
| --- | --- | --- | --- | --- | --- | --- |
| ICH | CNN-RNN (Sli-Lab) | 0.96 | 0.86 | 0.99 | 0.91 | 0.99 |
|  | 2D VGG16 | 0.95 | 0.86 | 0.98 | 0.90 | 0.98 |
|  | GoogleNet | 0.94 | 0.83 | 0.98 | 0.88 | 0.98 |
| CPH | CNN-RNN (Sli-Lab) | 0.95 | 0.87 | 0.97 | 0.87 | 0.98 |
|  | 2D VGG16 | 0.95 | 0.83 | 0.98 | 0.86 | 0.98 |
|  | GoogleNet | 0.95 | 0.82 | 0.98 | 0.86 | 0.98 |
| SAH | CNN-RNN (Sli-Lab) | 0.90 | 0.54 | 0.97 | 0.64 | 0.93 |
|  | 2D VGG16 | 0.82 | 0.20 | 0.93 | 0.25 | 0.78 |
|  | GoogleNet | 0.82 | 0.21 | 0.93 | 0.27 | 0.79 |
| EDH | CNN-RNN (Sli-Lab) | 0.99 | 0.61 | 1.00 | 0.68 | 0.97 |
|  | 2D VGG16 | 0.99 | 0.55 | 1.00 | 0.64 | 0.93 |
|  | GoogleNet | 0.99 | 0.50 | 1.00 | 0.62 | 0.93 |
| SDH | CNN-RNN (Sli-Lab) | 0.97 | 0.78 | 0.98 | 0.78 | 0.97 |
|  | 2D VGG16 | 0.96 | 0.59 | 0.99 | 0.72 | 0.96 |
|  | GoogleNet | 0.96 | 0.55 | 1.00 | 0.70 | 0.96 |
| IVH | CNN-RNN (Sli-Lab) | 0.95 | 0.70 | 0.99 | 0.78 | 0.96 |
|  | 2D VGG16 | 0.84 | 0.20 | 0.92 | 0.22 | 0.74 |
|  | GoogleNet | 0.85 | 0.23 | 0.93 | 0.25 | 0.75 |

**References**

1 Cho K, Merrienboer BV, Bahdanau D, Bengio Y (2014) On the Properties of Neural Machine Translation: Encoder-Decoder Approaches. Eighth Workshop on Syntax, Semantics and Structure in Statistical Translation, Doha, Qatar

2 Paszke A, Gross S, Lerer A (2017) Automatic differentiation in PyTorch. 31st Conference on Neural Information Processing Systems, Long Beach, CA, USA

3 Kingma DP, Ba J (2015) Adam-A Method for Stochastic Optimization. International Conference on Learning Representations 2015 (ICLR 2015), San Diego, CA

4 Simonyan K, Zisserman A (2014) Very Deep Convolutional Networks for Large-Scale Image Recognition. arXiv preprint arXiv: 1409.1556

5 Szegedy C, Liu W, Jia Y et al (2015) Going deeper with convolutions. 2015 IEEE Conference on Computer Vision and Pattern Recognition (CVPR):1-9

6 Prevedello LM, Erdal BS, Ryu J et al (2017) Automated Critical Test Findings Identification and Online Notification System Using Artificial Intelligence in Imaging. Radiology 285 3:923-931
